# Serum Survival of *Vibrio vulnificus*: Role of Genotype, Capsule, Complement, Clinical Origin, and *in Situ* Incubation

**DOI:** 10.3390/pathogens3040822

**Published:** 2014-10-03

**Authors:** Tiffany C. Williams, Mesrop Ayrapetyan, Heather Ryan, James D. Oliver

**Affiliations:** 1 Department of Biology, University of North Carolina at Charlotte, Charlotte, NC 28223, USA; E-Mails: tcwilli1@uncc.edu (T.C.W.); mesrop.ayrapetyan@gmail.com (M.A.); hr319@nova.edu (H.R.); 2 Nicholas School of the Environment, Duke University Marine Laboratory, Beaufort, NC 28516, USA

**Keywords:** *in situ* incubation, wound infections, capsule

## Abstract

Virulence of the human pathogen, *V. vulnificus*, is associated with encapsulation, serum complement resistance, and genotype. The C-genotype of this bacterium is correlated (>90%) with virulence and with isolation source (clinical settings). E-genotype strains are highly correlated with environmental isolation (93%) but appear less virulent. In this study, we characterized the importance of genotype, encapsulation, serum complement, and *in situ* exposure to estuarine water on the survival of the two genotypes in human serum. Results confirmed the superior ability of C-genotype strains to survive exposure to human serum, as well as the significance of complement, and revealed that lack of capsule allowed serum killing of both C- and E-genotypes. Cells incubated *in situ* responded similarly to cells incubated *in vitro* with the exception of E-environmental strains. Interestingly, our studies found that those cells of the E-genotype, typically considered non-pathogenic, which were isolated from wound infections demonstrated serum survival similar to that of virulent, C-genotype, strains.

## 1. Introduction

*Vibrio vulnificus* exists in estuaries across the globe, and is associated with a variety of aquatic organisms, particularly bivalves such as the eastern oyster, *Crassostrea virginica*. Ingestion of raw or undercooked oysters carrying *V. vulnificus* can lead to rapid and severe septicemia, multi-organ failure, necrotizing fasciitis, and in some cases death [1,2,3]. Indeed, *V. vulnificus* accounts for ~95% of all seafood related deaths in the U.S. and has the highest case fatality rate (>50%) of any foodborne pathogen [4,5].

While most bacterial pathogens display a single mode of transmission, *V. vulnificus* has an alternate route of infection via introduction into an open wound [6]. Wound infections typically result from a wound inflicted by handling shellfish or other recreational activities in coastal environments and carry a mortality rate of *ca.* 25% [2].

Not all strains of this bacterium are equally pathogenic; of the three known biotypes of *V. vulnificus*, biotype 1 represents the majority of strains that can result in human disease upon exposure. This biotype can be further subdivided into two genotypes, aptly named for their most common sources of isolation. “C” (clinical) genotypes are typically pathogenic, and 93% of isolates from clinical settings are of this genotype [7]. Conversely, “E” (environmental) genotypes are generally non-pathogenic, and 90% percent are isolated from environmental settings, including oysters, sediment, and water samples [7]. These two genotypes can be distinguished using a simple and rapid polymerase chain reaction, in which one of two virulence correlated alleles, *vcgE* or *vcgC*, is amplified [8].

Although C- and E-genotypes significantly correlate with isolation source, it is becoming increasingly apparent that a subset of E-genotypes has acquired the ability to cause human disease, with most isolates being implicated in wound infections. This is of particular medical concern considering the documented increase in the number of *V. vulnificus* wound infections and subsequent deaths as a result of climate change [9].

The pathogenicity of this bacterium is believed to arise from several putative virulence factors, including the production of iron-binding siderophores, exoenzymes, and capsular polysaccharides, as well as the ability to survive exposure to human serum [1,10,11,12]. While most of the proposed virulence factors remain unconfirmed, the production of capsular polysaccharide (CPS) is highly correlated with pathogenicity [1,10,13,14,15]. In fact, previous research has shown that the LD_50_ of the encapsulated, “opaque” strains is as low as a single cell [13] whereas non-encapsulated, “translucent” strains of *V. vulnificus* display little or no virulence [13,14].

Our lab and others have previously reported on the ability of some *V. vulnificus* strains to survive exposure to human serum [12,16,17,18]. Both the C- and E- genotypes of *V. vulnificus* typically produce CPS, a trait known to aid in defense against serum, yet our lab has found the E-genotypes to show greater susceptibility when exposed to human serum than C-genotypes [16]. A variety of suggestions for the mechanisms of this phenomenon have been offered, the most significant of which may be differences in expression of the siderophore gene, *viuB* or capsule switching. Previous research from our lab indicated that C-genotype cells, all of which were *viuB* positive, showed significantly higher serum survival than E-genotype strains, most of which lacked this siderophore-encoding gene [16]. Our studies have also indicated that E-genotype cells more frequently revert to the non-encapsulated, translucent phenotype than cells of the C-genotype [19], possibly rendering them more susceptible to the bactericidal effects of serum.

The intent of the present study was to further characterize the degree of serum resistance demonstrated by the two genotypes of *V. vulnificus,* with particular attention to clinically isolated E-genotypes. We also investigated the role of capsule in serum survival, employing strains that have undergone a permanent mutation in the *wzb* gene within the CPS operon. A unique aspect of this study involved exposing cells to environmental conditions to induce the natural physiology of *V. vulnificus* cells prior to introduction into human serum. This aspect was accomplished by subjecting the cells to 24 h of *in situ* incubation in estuarine water using membrane diffusion chambers.

## 2. Results and Discussion

### 2.1. Role of Genotype and Isolation Source in Serum Survival

**Figure 1:**
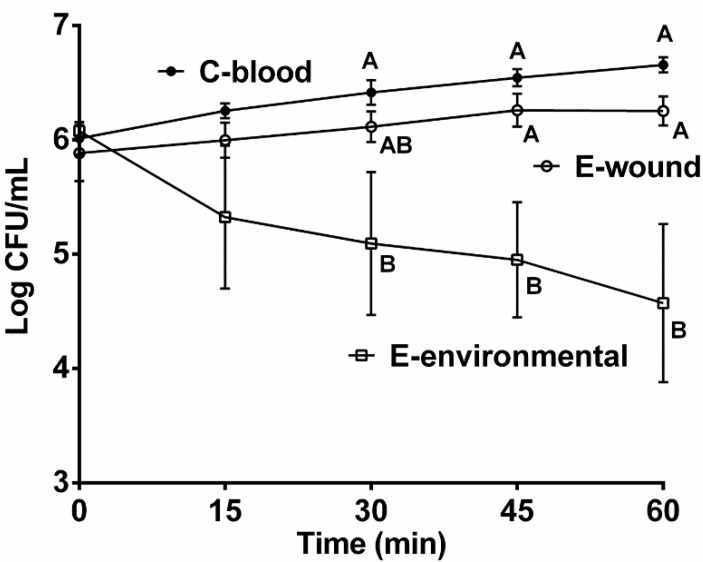
Role of genotype and isolation source on survival in human serum. Survival of clinically isolated C-genotypes (MO6-24, CMCP6, C7184, YJ016; closed circles), E-genotype wound isolates (E64MW, LSU2098, LSU1657, LSU549; open circles) and environmentally isolated E-genotypes (JY1305, JY1701, ENV1, SS108-A3A; open squares), cells exposed to human serum for 60 min. Error bars represent the standard error of the mean for four strains with three replicates each. Different letters indicate statistically significant differences (two-way ANOVA).

Examination of four clinically isolated C-genotype and four environmentally isolated E-genotype strains revealed a significant difference in their ability to survive in human serum (Figure 1). While C-genotype strains exhibited total survival and even growth, exposure to serum was inhibitory to E-genotype growth, supporting results previously reported [16]. This difference was particularly evident after 60 min of incubation.

While C-genotypes predominate in septicemia cases, a considerable number of E-genotypes have been isolated from wound infections. This finding has prompted further interest into E-genotype strains, *i.e.*, do all E-genotypes have the ability to cause wound infections or is there a subset of E-genotypes that have unique virulence factors or share virulence factors with C-genotypes? Additionally, *V. vulnificus* wound infections associated with recreational water activities have become more prevalent in both the U.S. and Europe likely as a result of warming water temperatures [9,20,21,22]. We therefore examined if E-genotype strains isolated from wound infections differ in human serum sensitivity compared to non-clinical, environmentally isolated E-genotypes. Unlike environmental E-genotypes, wound isolates resisted the bacteriocidal effects of human serum at a rate similar to that of C-genotypes (Figure 1). Interestingly, a recent comparative genome analysis [23] revealed that the E-genotype wound isolate, E64MW, shares 43 genes with three C-genotype blood isolates (CMCP6, YJ016, and M06-25) which were absent in the two non-clinical E-genotypes (JY1305 and JY1701). From these results we suggest that a subgroup of E-genotypes have acquired mechanisms to successfully colonize and infect the human host although further investigation is required to more fully understand the relationship between C-genotypes and this subset of E-genotypes.

### 2.2. Role of Capsule in Serum Survival

**Figure 2:**
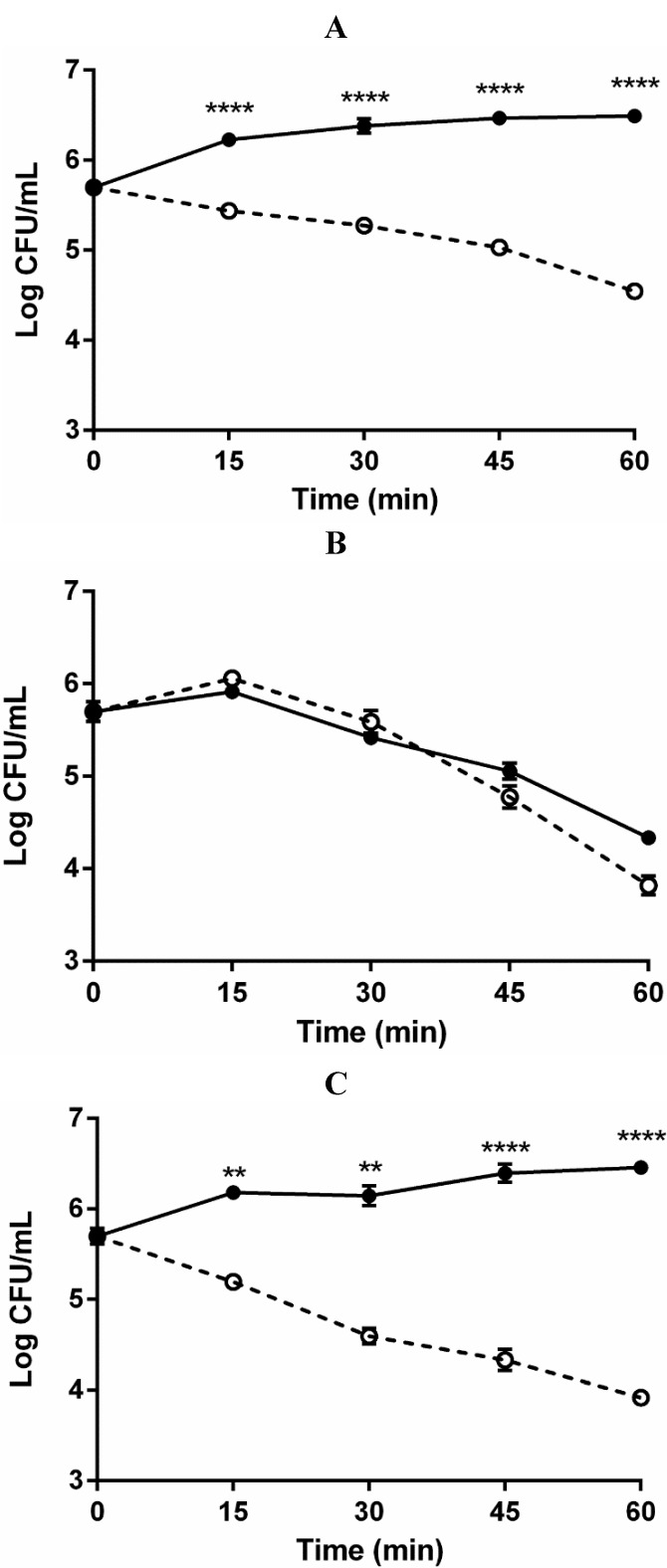
Role of capsular polysaccharide in survival of *V. vulnificus* exposed to human serum. (**A**) Survival of opaque and translucent clinical C-genotype (C7184/Op, closed circles; C7184/Tr, open circles); (**B**) Opaque and translucent environmental E-genotype (JY1701/Op, closed circles; JY1701/Tr, open circles); (**C**) Opaque and translucent clinical E-genotype (LSU1657, closed circles; LSU1657/Tr, open circles) exposed to human serum for up to 1 hour. Error bars represent the standard error of the mean for three replicates per strain. Two-Way ANOVA with Bonferroni *post hoc* test (** *p* < 0.01; **** *p* < 0.0001).

We also analyzed the effect of capsule on serum survival by both C- and E-genotypes. All capsular polysaccharide mutants employed appeared phenotypically identical and possess genetic determinants affecting the functionality of the group 1 CPS operon which directs CPS biosynthesis and transport [14,24,25]. In contrast to the encapsulated clinical strains (C and E genotypes) which exhibited population growth, the non-encapsulated strains of both genotypes underwent more than a 1-log decrease in culturability within 60 min (Figure 2).

Culturability of the non-encapsulated environmental E-genotype strain decreased nearly 2-logs. Using the standard method for visual determination of encapsulation [13,15], none of the non-encapsulated mutants exhibited capsule when plated whereas all parent strains produced the “opaque”, encapsulated colonial phenotype. Thus, the presence of CPS appears to play a significant role in serum survival regardless of genotype or clinical/environmental source (Figure 2). This is likely due to the resistance that negatively charged CPS imparts against antimicrobial components present in human serum, or to shifts in osmolarity [26].

### 2.3. Role of Complement in Serum Survival

**Figure 3:**
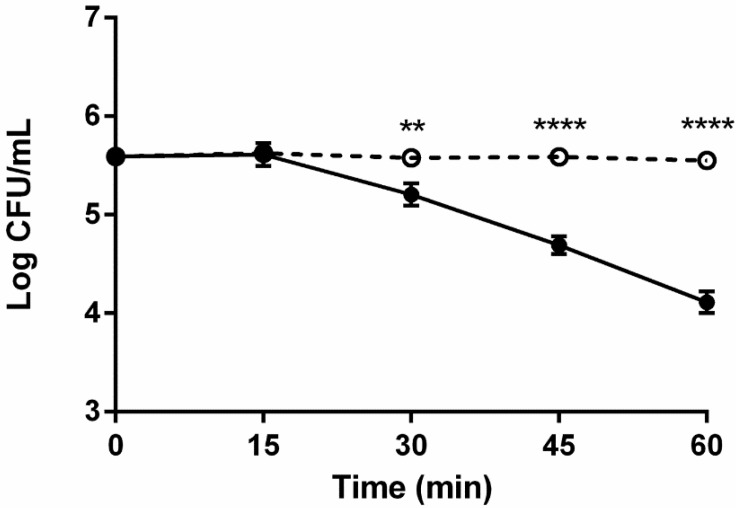
Role of complement in bactericidal activity of serum against translucent strains of *V. vulnificus.* Effect of complement active serum on translucent strains (C7184/Tr; LSU1657/Tr; and JY1701/Tr) in complement active (closed circles) or complement inactive (open circles) serum. Error bars represent the standard error of the mean for three replicates per strain. Asterisks indicate statistically significant differences. Two-Way ANOVA with Bonferroni *post hoc* test (** *p* < 0.01; **** *p* < 0.0001).

Inactivated serum allowed for more than 1-log greater survival of non-encapsulated strains compared to survival in active serum (Figure 3). These results indicate that inactivation of bacteriocidal components, including complement proteins, allows for survival and even growth of non-encapsulated strains, regardless of genotype.

### 2.4. Effect of in situ Incubation on Serum Survival

**Figure 4:**
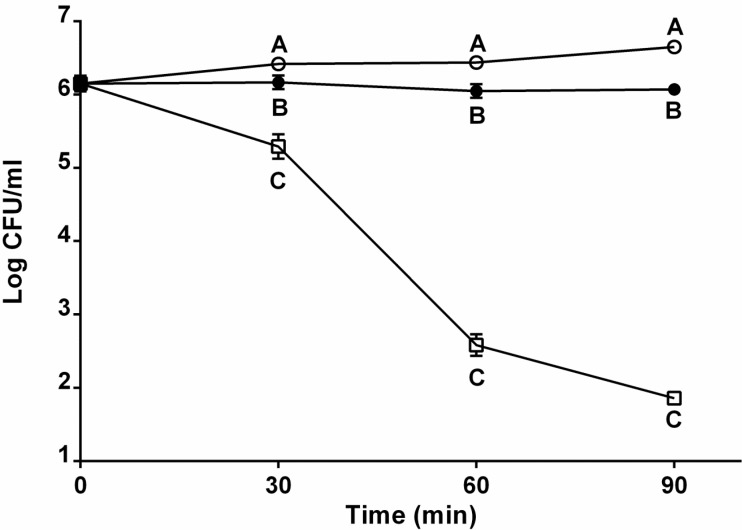
Serum survival of *V. vulnificus* genotypes and isolate types following *in situ* incubation. Survival of clinically isolated C-genotypes (CMCP6, YJ016; open circles), clinically isolated E-genotypes (LSU2098, E64MW; closed circles), and environmentally isolated E-genotypes (JY1305, ENV1; squares) in human serum for up to 90 min after incubation *in situ* (estuarine waters). Error bars represent the standard error of the mean for two strains and three replicates. Different letters indicate statistically significant differences (two-way ANOVA).

To determine if cells of *V. vulnificus* present in the natural environment respond to human serum in a manner similar to what we observed for *in vitro* grown cells, we exposed cells to *in situ* conditions by placing them in an estuary for 24 h prior to their exposure to human serum. This was accomplished employing membrane diffusion chambers that house the cells while allowing them to be exposed to salinity, temperature, nutrient, viruses and other dissolved matter fluctuations naturally present in such environments. As seen with our *in vitro* studies, both clinically isolated C-genotype and wound E-genotype isolates maintained serum resistance (Figure 4) whereas environmental E-genotype strains were more susceptible to serum relative to survival *in vitro* (Figure 1 and Figure 4).

Thus, little difference in survival was detected between cells incubated *in situ* and *in vitro* prior to exposure to serum, with the exception of E-environmental strains which displayed a weaker resistance to human serum following *in situ* incubation. This finding suggests that the environmental conditions cells experience in estuarine waters, such as fluctuating salinity, temperature, and nutrient availability, may alter the potential for serum survivability. Differences in expression of the hemolysin, *vvhA*, had previously been observed by our lab for cells of the two *V. vulnificus* genotypes incubated *in situ* [27].

## 3. Materials and Methods

### 3.1. Bacterial Strains and Growth Conditions

Table 1 lists the strains that were used in this study along with genotype, source of isolation, and capsule phenotype. The translucent strains used in this study, originally isolated and identified by our lab, underwent a spontaneous mutation of the *wzb* gene resulting in permanent loss of its ability to produce capsular polysaccharide. These strains are referred to as TR2 strains as previously described [24] and were genetically confirmed to lack the *wzb* gene via PCR while still possessing the flanking *wza* and wzb genes.

Bacterial cultures were taken from freezer stocks and grown overnight in heart infusion (HI) broth at 30 °C with shaking. For *in vitro* studies, overnight cultures were diluted in fresh HI broth at a 1/100 (v/v) ratio and grown to logarithmic phase (OD_610_ 0.15–0.25).

### 3.2. In Situ Incubations

Membrane diffusion chambers used in this study were originally designed by McFeters and Stuart [28,29]. These consist of two 76 mm, 0.2 µm hydrophilic polycarbonate filters (Midland Scientific Inc.; cat# 1220891) sandwiched between three doughnut shaped sections of Plexiglas. Using this apparatus, 25 mL of bacterial culture at a final cell concentration of *ca.* 10^4−5^ CFU/mL was aseptically injected into each autoclaved chamber which were then deployed into Calico Creek, an estuarine water body located in Beaufort, North Carolina. Water temperatures at the site of deployment were typically 20–25 °C with salinities of 15–34 ppt. After 24 h of incubation, the chambers were retrieved and the cells aseptically removed for serum studies.

**Table 1:** List of *V. vulnificus* strains used in this study including their respective genotypes, source of isolation, and colony opacities (capsule presence).

Strain Name	Genotype	Isolation Source	Opacity
CMCP6	C	Human Blood	Opaque
YJ016	C	Human Blood	Opaque
MO6-24	C	Human Blood	Opaque
C7184	C	Human Blood	Opaque
C7184/Tr^a^	C	Spontaneous CPS mutant	Translucent
LSU2098	E	Human wound	Opaque
E64MW	E	Human wound	Opaque
LSU549	E	Human wound	Opaque
LSU1657	E	Human wound	Opaque
LSU1657/Tr^a^	E	Spontaneous CPS mutant	Translucent
ENV1	E	Water	Opaque
SS108-A3A	E	Oyster	Opaque
JY1305	E	Oyster	Opaque
JY1701	E	Oyster	Opaque
JY1701/Tr^a^	E	Spontaneous CPS mutant	Translucent

^a^ Isogenic CPS mutants lacking the *wzb* gene of the CPS operon, referred to as a TR2 genotype by Chatzidaki-Livanis *et al.* [24].

### 3.3. Human Serum Exposure

Pooled male human serum (MP Biomedicals, Santa Ana, CA, USA) was used for all studies. To determine the significance of bacteriocidal components, including the complement cascade, in serum sensitivity, serum was heat-inactivated by incubation of the serum at 56°C for 30 min. *In vitro* studies were adapted from Bogard and Oliver [16]. To achieve a final cell concentration of *ca.* 10^5−6^ cells/mL serum, log-phase cells (12 µL) were inoculated into 788 µL serum, or in the case of *in situ* incubation, 100 µL of cells were added to 1 mL of serum. In all cases cells were incubated in serum at 37°C for up to 2 h, with culturability assessed at 15 or 30 min time intervals by serially diluting into PBS followed by plating onto HI agar to determine CFU/mL after 24 h incubation at 30°C.

### 3.4. Statistical Analysis

Each experiment was performed with at least three replicates per strain. Log transformed data were analyzed using GraphPad Prism (v. 5.0; GraphPad Software Inc. San Diego, CA, USA). Statistical analyses were performed using one and two-way analyses of variance (ANOVA) followed by Bonferroni *post hoc* test for multiple comparisons. Significance was determined using a 95% confidence interval. 

## 4. Conclusions

The findings reported here support previous studies and demonstrate that genotype and capsular polysaccharide significantly impact *V. vulnificus* serum survivability and likely pathogenicity, and are therefore important virulence determinants for this organism. However, E-genotype strains isolated from wound infections were found to exhibit serum resistance similar to that of the clinically isolated C-genotypes, and this resistance was maintained regardless of *in vitro* or *in situ* incubation prior to exposure to serum. Thus, while genotype largely correlates with source of isolation, further genetic distinctions within E-genotypes are necessary to predict pathogenic potential.
